# Case Report: Hemodynamic Instability Caused by Splenic Rupture During Video-Assisted Thoracoscopic Lobectomy

**DOI:** 10.3389/fsurg.2022.900396

**Published:** 2022-04-22

**Authors:** Chun-tong Liu, Ting-ting Wu, Yun-ying Ding, Jin-long Lin, Shuang Zhou, Hong Liu, Fu-hai Ji, Ke Peng

**Affiliations:** ^1^ Department of Anesthesiology, First Affiliated Hospital of Soochow University, Suzhou, Jiangsu, China; ^2^ Institute of Anesthesiology, Soochow University, Suzhou, Jiangsu, China; ^3^ Department of Anesthesiology and Pain Medicine, University of California Davis Health System, Sacramento, CA, United States

**Keywords:** video-assisted thoracoscopic surgical, splenic rupture, hemodynamic instability, anesthesia management, urgent splenectomy

## Abstract

**Background:**

Video-assisted thoracoscopic surgery (VATS) has been widely performed for patients with lung cancer. Splenic rupture after VATS lung procedures is a very rare and serious event.

**Case Presentation:**

We reported a case with hemodynamic instability after left lower VATS lobectomy. There was no evidence of diaphragmatic injury during the surgery. Computed tomography (CT) showed spleen injury and large amount of fluid in the abdominal cavity. Emergent laparotomy was performed, and splenic rupture was diagnosed. The patient underwent splenectomy, with two lacerations at the diaphragmatic surface of the spleen. The patient did well postoperatively and was discharged from the hospital on postoperative day 5.

**Conclusion:**

There are few similar cases reported in the literature. Persistent hemodynamic instability due to the rupture of spleen is life-threatening. In the situation of unexplained hypotension during VATS procedures (especially left-sided approaches), the possibility of splenic injury and rupture should be considered. Abdominal ultrasonography and/or CT examinations should be carried out for prompt diagnosis and treatment of such rare complication.

## Introduction

Video-assisted thoracoscopic surgical procedures (VATS) are widely performed for treatment of patients with lung cancer. Growing evidence have shown the advantages of VATS lobectomy over thoracotomy (1). Splenic rupture after VATS lung procedures is a very rare but catastrophic event. Here, we reported a case with persistent hemodynamic instability after VATS lobectomy. After the splenic rupture was eventually diagnosed, the patient received splenectomy. This report adheres to the CARE guidelines, with completed CARE checklist in Supplementary File S1.

## Case Report

A 67-year-old female (165 cm height and 68 kg weight) was admitted to our hospital for left lower lung adenocarcinoma and underwent left lower VATS lobectomy. The patient was at American Society of Anesthesiologists physical status II with no major comorbidities. Hemoglobin (Hb) was 11.7 g/dL, and platelet count was 119 × 10^9^/L. Preoperative laboratory tests revealed no abnormalities in coagulation function or other parameters.

Pre-induction non-invasive cuff blood pressure was 142/78 mmHg, heart rate was 72 beats/min, and peripheral oxygen saturation was 97%. The patient received an opioid-free anesthesia regimen. Dexmedetomidine 0.6 µg/kg was administered over 10 min. An arterial line was inserted into the right radial artery for measuring arterial blood pressure. Esketamine 0.3 mg/kg, propofol 2 mg/kg, and rocuronium 0.6 mg/kg were used for induction. A left-sided double-lumen endobronchial tube was used for intubation and confirmed by auscultation, capnography, and fiberoptic bronchoscopy. One-lung ventilation (tidal volume 350–400 mL, respiratory rate 12–18 breaths/min, I/E = 1:2, 60%–100% oxygen) was started. Anesthesia was maintained with sevoflurane 1%–3% inhalation, dexmedetomidine infusion 0.3–1.0 µg/kg/h, and intermittent esketamine boluses 0.1–0.2 mg/kg. Dexamethasone 5 mg and ondansetron 8 mg were given for prophylaxis of postoperative nausea and vomiting.

The surgical procedure started at 16:32. The patient’s hemodynamics remained stable for about 3 h into surgery. At 19:30, patient’s blood pressure dropped from 130/75 mmHg to 88/51 mmHg. Phenylephrine 50 µg was administered intravenously to treat the hypotension, and dexmedetomidine infusion was also stopped. The anesthesiologist discussed the situation with the thoracic surgeons. They checked the surgical field and found no obvious bleeding. Using intermittent boluses of phenylephrine and/or ephedrine, blood pressure was maintained within 100–130/60–80 mmHg. The surgery was completed at 21:00. Intraoperative fluid infusion consisted of lactate Ringer’s solution 1,000 mL and gelatin solution 500 mL. Intraoperative blood loss was approximately 300 mL. Intraoperative urine output was 400 mL.

At the end of the surgery, the arterial blood gas and electrolyte analyses showed that pH = 7.357, pCO_2 _= 41.1 mmHg, pO_2 _= 294 mmHg, Hb = 8.2 g/dL, Ca^2+ ^= 1.07 mmol/L, and lactate = 1.5 mmol/L (Table 1). At 21:30, the double-lumen endobronchial tube was removed after the patient fully awaked. Supplemental oxygen at 2 L/min was delivered via a nasal catheter. Shortly thereafter, her blood pressure dropped from 120/70 mmHg to 85/46 mmHg, and heart rate was 80 beats/min. 10% calcium gluconate 10 mL was administered intravenously. At 22:22, the Hb was 8.4 g/dL (Table 1). Blood pressure still fluctuated within 70–100/50–65 mmHg. The surgeons rechecked the drainage and confirmed no bleeding in the chest. At this moment, the patient complained of chest tightness. Myocardial ischemia and cardiogenic shock were suspected. The electrocardiogram showed no abnormality in the ST segment, and high-sensitivity troponin T results did not support myocardial injury. Gelatin anaphylaxis was also considered in the differential diagnosis, and methylprednisolone 40 mg and intermittent low-dose epinephrine 10–20 µg were given intravenously. Bedside chest X-ray ruled out pneumothorax, intrathoracic bleeding, or other intrathoracic abnormalities.

**Table 1:** Arterial blood gas and electrolyte analyses.

Parameter	Timepoint				
	21:30	22:22	00:33	01:59	02:04
pH	7.357	7.351	7.314	7.294	7.284
pCO_2_ (mmHg)	41.1	42.3	38.8	33.7	35.4
pO_2_ (mmHg)	294	66.1	73.6	96.9	93.8
Hb (g/dL)	8.2	8.4	8.8	6.8	7.0
sO_2_ (%)	99.9	94.2	96.2	98.4	98.1
O_2_Hb (%)	98.4	92.0	93.7	95.7	95.7
K^+^ (mmol/L)	3.5	3.7	3.9	3.2	3.2
Na^+^ (mmol/L)	141	140	139	141	141
Ca^2+^ (mmol/L)	1.07	1.19	1.11	1.06	1.11
Cl^-^ (mmol/L)	112	111	110	112	111
Glu (mmol/L)	7.6	10.4	15.0	12.8	13.1
Lac (mmol/L)	1.5	1.6	3.8	6.3	6.9
Hct (%)	25.2	25.7	27.1	20.9	21.4
p50 (mmHg)	27.57	23.98	22.37	28.68	29.14
HCO_3_^−^ std (mmol/L)	22.5	22.6	19.4	16.8	17.0
HCO_3_^−^ (mmol/L)	23.1	23.4	19.7	16.3	16.8
BE(B) (mmol/L)	−2.4	−2.2	−6.5	−10.2	−9.9

Because of sustained hemodynamic instability, a central venous catheter was placed in the internal jugular vein under ultrasonography, for central venous pressure (CVP) monitoring, fluid resuscitation, and infusion of vasopressors (norepinephrine and dopamine). In addition, cardiac index (CI) and stroke volume variation (SVV) were monitored using the FloTrac/Vigileo system. At 23:55, the monitor showed that CVP = 4 mmHg, CI = 2.6 L/min/m^2^, and SVV = 19%. Based on these, hypovolemia was considered. Lactate Ringer’s solution 500 mL, hydroxyethyl starch 500 mL, erythrocytes 3 units, and fresh frozen plasma 500 mL were given. At 00:33, Hb was 8.8 g/dL, and lactate was 3.8 mmol/L (Table 1). Blood pressure was maintained within 100–120/60–80 mmHg, and heart rate was 80–100 beats/min, with a small-dose epinephrine infusion (0.03–0.05 µg/kg/min).

**Figure 1:**
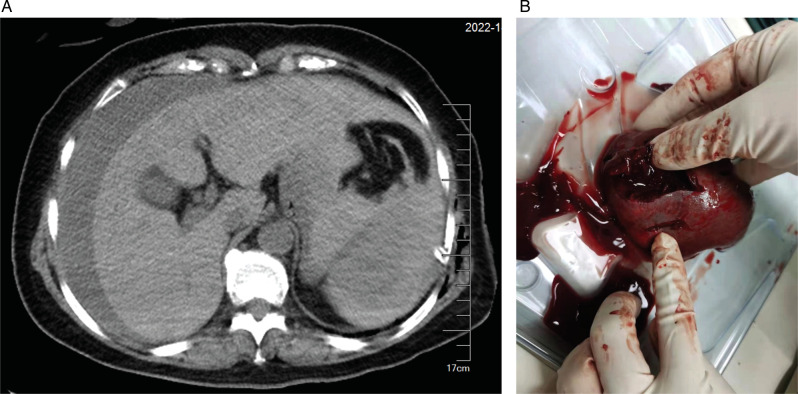
Images of abdominal computed tomography and ruptured spleen. (**A**) Abdominal computed tomography showing splenic hematoma and large amount of free intraperitoneal fluid. (**B**) Two lacerations of the ruptured spleen.

At 01:59, blood analysis showed that pH decreased to 7.294, Hb dropped to 6.8 g/dL, and lactate increased to 6.3 mmol/L (Table 1). These results were confirmed by an additional test at 02:04. The patient reported upper abdominal pain, and her abdomen was more distended than before. The bedside abdominal ultrasonography showed free intraperitoneal fluids (images not captured). Abdominal computed tomography (CT) confirmed splenic injury and large amount of fluid in the abdominal cavity (Figure 1A). Emergent laparotomy was performed, and splenic rupture was diagnosed. The patient underwent splenectomy, with two lacerations on the diaphragmatic surface of the spleen (Figure 1B). The hemodynamic changes and treatment for hypotension of this patient were summarized in Table 2. After surgery, the patient was admitted to the thoracic intensive care unit. The patient did well postoperatively. She was transferred to the general ward at postoperative day 1 and discharged from the hospital on postoperative day 5.

**Table 2:** Hemodynamic changes and treatment for hypotension.

Parameter	Timepoint									
	Baseline (16:00)	Start of VATS (16:32)	3 h into surgery (19:30)	End of VATS (21:00)	Tracheal extubation (21:30)	1 h post-extubation (22:30)	3 h post-extubation (00:30)	4.5 h post-extubation (02:00)	Start of laparotomy (03:20)	End of splenectomy (05:00)
HR (beats/min)	72	69	78	70	87	75	98	101	93	70
ABP (mmHg)	142/79	143/78	88/51	105/60	89/47	81/46	102/61	120/50	83/41	104/58
CVP (mmHg)	/	/	/	/	/	/	6	3	4	8
CI (L/min/m^2^)	/	/	/	/	/	/	2.7	2.6	2.4	2.3
SVV (%)	/	/	/	/	/	/	19	7	17	11
Vasopressors (type and dosage)	/	/	Phenylephrine 50 µg boluses	Boluses of phenylephrine/ ephedrine	Phenylephrine 50–100 µg boluses	Epinephrine 10–20 µg	Norepinephrine, dopamine, or epinephrine	Epinephrine 0.03–0.05 µg/kg/min	Phenylephrine 50–100 µg boluses	/
Other treatment	/	/	Dexmedetomidine stopped	Intravenous fluids	10% calcium gluconate 10 mL	Methylprednisolone 40 mg	CVC placed, erythrocytes 3 u, FFP 500 mL	Abdominal CT	Erythrocytes 7 u, FFP 500 mL, cryoprecipitate 3 u	Vitamin K1 20 mg, PCC 400 u, fibrinogen 1 g
Possible causes for hypotension	/	/	Hypovolemia, surgical bleeding	Hypovolemia	Postoperative bleeding	Myocardial ischemia, cardiogenic shock, and/or anaphylaxis	Hypovolemia and/or anaphylaxis	Splenic injury, intraperitoneal hemorrhage	Intraperitoneal hemorrhage, hemorrhagic shock	Splenic rupture

*HR, heart rate; ABP, arterial blood pressure; CVP, central venous pressure; CI, cardiac index; SVV, stroke volume variation; VATS, video-assisted thoracoscopic surgery; CVC, central venous catheter; CT, computed tomography; PCC, prothrombin complex concentrate; FFP, fresh frozen plasma.*

## Discussion

Splenic rupture is an uncommon and serious complication of VATS procedures. Only four similar cases were previously reported in the literature (2–5). Among them, one patient was managed with angiographic arterial embolization of the spleen, and the other three patients received splenectomy. Our patient also underwent splenectomy. In all reported cases including our patient, there was no evidence of diaphragmatic laceration during the surgical procedures, and radiographic examinations also showed that the diaphragm was intact. The injury to the spleen may be caused by transdiaphragmatic blunt trauma, which was possibly associated with the positioning of thoracoscopic ports and surgical manipulations. In the absence of diaphragmatic injury during surgery, early diagnosis of splenic rupture is usually difficult. In our case, after excluding hypovolemic shock and surgical bleeding, the cause of hemodynamic instability was considered as cardiogenic shock or anaphylactic shock, and eventually back to hypovolemic shock. We continuously monitored the patient’s hemodynamic fluctuations and maintained her blood pressure with vasopressors and volume therapy; however, to some extent, the diagnosis of splenic rupture of this case was delayed.

From this case, we can learn that, in the situation of unexplained hemodynamic instability during the VATS procedures, the possibility of splenic injury and rupture should be considered. For patients with clinical features of hemodynamic instability and abdominal pain after VATS, abdominal ultrasound and/or CT examinations should be carried out as soon as possible for prompt diagnosis and treatment.

## Data Availability

The original contributions presented in the study are included in the article/Supplementary Material, further inquiries can be directed to the corresponding authors.

## References

[1] Boffa DJ Dhamija A Kosinski AS Kim AW Detterbeck FC Mitchell JD Fewer complications result from a video-assisted approach to anatomic resection of clinical stage I lung cancer. J Thorac Cardiovasc Surg. (2014) 148(2):637–43. 10.1016/j.jtcvs.2013.12.045 24529729

[2] Flores RM Ihekweazu U Dycoco J Rizk NP Rusch VW Bains MS Video-assisted thoracoscopic surgery (VATS) lobectomy: catastrophic intraoperative complications. J Thorac Cardiovasc Surg. (2011) 142(6):1412–7. 10.1016/j.jtcvs.2011.09.028 22014713

[3] Forti Parri SN Guiducci GM Domanico A Tugnoli G. Splenic rupture after videothoracoscopic procedure: an unusual complication conservatively managed. J Thorac Cardiovasc Surg. (2014) 148(5):e236–7. 10.1016/j.jtcvs.2014.08.004 25167983

[4] Zhang Z. A care report of splenic rupture with subcapsular hematoma after thoracoscopic surgery. Chin J Endosc. (2001) 5(7):27. [Article in Chinese]. 10.3969/j.issn.1007-1989.2001.05.064

[5] Li J Wang L. Anesthesia management for a patient with splenic rupture during thoracoscopic surgery. J Clin Anesthesiol. (2018) 34(3):312. [Article in Chinese]. 10.12089/jca.2018.03.026

